# Thermal Runaway of Nonflammable Localized High‐Concentration Electrolytes for Practical LiNi_0.8_Mn_0.1_Co_0.1_O_2_|Graphite‐SiO Pouch Cells

**DOI:** 10.1002/advs.202204059

**Published:** 2022-09-08

**Authors:** Yu Wu, Xuning Feng, Min Yang, Chen‐Zi Zhao, Xiang Liu, Dongsheng Ren, Zhuang Ma, Languang Lu, Li Wang, Gui‐Liang Xu, Xiangming He, Khalil Amine, Minggao Ouyang

**Affiliations:** ^1^ School of Materials Science and Engineering Beijing Institute of Technology Beijing 100081 P. R. China; ^2^ State Key Laboratory of Automotive Safety and Energy Tsinghua University Beijing 100084 P. R. China; ^3^ Institute of Nuclear and New Energy Technology Tsinghua University Beijing 100084 P. R. China; ^4^ Chemical Sciences and Engineering Division Argonne National Laboratory Lemont IL 60439 USA

**Keywords:** localized high concentration electrolyte, NMC811|Gr‐SiO, nonflammable, practical pouch‐type cells, thermal runaway

## Abstract

With continuous improvement of batteries in energy density, enhancing their safety is becoming increasingly urgent. Herein, practical high energy density LiNi_0.8_Mn_0.1_Co_0.1_O_2_|graphite‐SiO pouch cell with nonflammable localized high concentration electrolyte (LHCE) is proposed that presents unique self‐discharge characteristic before thermal runaway (TR), thus effectively reducing safety hazards. Compared with the reference electrolyte, pouch cell with nonflammable LHCE can increase self‐generated heat temperature by 4.4 °C, increase TR triggering temperature by 47.3 °C, decrease the TR highest temperature by 71.8 °C, and extend the time from self‐generated heat to triggering TR by ≈8 h. In addition, the cell with nonflammable LHCE presents superior high voltage cycle stability, attributed to the formation of robust inorganic‐rich electrode–electrolyte interphase. The strategy represents a pivotal step forward for practical high energy and high safety batteries.

## Graphical abstract

This paper investigates the thermal runaway (TR) features of practical pouch‐type NMC811|Gr‐SiO cell using nonflammable localized high concentration electrolyte (LHCE). It should be noted that even with nonflammable LHCE, the cell still triggers TR and combustion, but the intrinsic safety can be improved. The paper calls for more safety verification at the level of cell, not just their flammability.

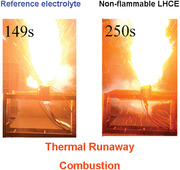

## 1 Introduction

The development of advanced lithium‐ion batteries (LIBs) in electric vehicles and smart grids requires high energy density and high safety.^[^ ^1^, ^2^, ^3^, ^4^ ^]^ To further increase the energy density, Ni‐rich layered oxides with the high capacity have recently become the mainstream cathodes for practical LIBs, whereas the anode material is changing from graphite to a mixture of SiO and graphite.^[^ ^5^, ^6^, ^7^, ^8^ ^]^ Owing to the highest energy density among current commercial LIBs, LiNi_0.8_Mn_0.1_Co_0.1_O_2_|Graphite‐SiO (NMC811|Gr‐SiO) cell has attracted tremendous interest in both academic and industrial research. However, the electrode materials with higher energy density generally have lower thermal stability, leading to severe safety hazards characterized thermal runaway (TR), especially at high working voltage.^[^ ^9^, ^10^, ^11^ ^]^ The TR is triggered by a series of exothermic reactions that simultaneously raise the temperature of LIBs.^[^ ^12^, ^13^, ^14^ ^]^ Thus, the removal of the triggering reactions involving high energy density electrodes is critical to achieve the safer LIBs.^[^ ^15^ ^]^

As the connection between cathode and anode materials of the LIBs, electrolyte plays a crucial role.^[^ ^16^, ^17^, ^18^ ^]^ However, conventional carbonate‐based electrolytes only has limited oxidation stability (≈4.3 V), hindering application to the high‐voltage cathodes.^[^ ^19^ ^]^ Beyond that, the interphase generated on alloy electrode cycled in conventional electrolyte is unstable and cannot suffer the huge volume changes.^[^ ^20^, ^21^ ^]^ More importantly, the violent exothermic reaction between ethylene carbonate (EC) solvent and NMC811 cathodes accelerates the triggering of TR,^[^ ^15^ ^]^ and its flammable nature increases the intensity of combustion. The conventional electrolyte using EC solvent has been adopted since the commercialization of LIBs until today, but it cannot keep up with the high voltage and high safety requirements of the next generation of high‐energy batteries.

In recent years, Zhang and co‐workers reported the localized high concentration electrolyte (LHCE) by adding nonsolvating diluent,^[^ ^22^, ^23^, ^24^, ^25^ ^]^ where the Li^+^ ion solvation structures similar to that of high concentration electrolyte can be maintained, which has become the most popular research topic.^[^ ^26^, ^27^, ^28^ ^]^ Moreover, the LHCE is beneficial to the formation of salt‐derived inorganic‐rich electrode–electrolyte interphase (EEI), which contributes to stabilize the electrode. Meanwhile, flame retarding electrolyte additive or cosolvent can be adopted to design nonflammable LHCE.^[^ ^29^, ^30^, ^31^, ^32^, ^33^, ^34^ ^]^ Despite these reported advantages may improve battery safety, it has only been tested on ignition and material‐level. There is still no report on safety of practical LIBs with nonflammable LHCE.

Herein, we investigate the safety features of practical pouch‐type high energy NMC811|Gr‐SiO cell employing nonflammable LHCE (1.0 m LiFSI/FEC:TEP:BTFE = 10:20:70 by volume), which can effectively stabilize NMC811 cathode and SiO anode interface at high operating voltage. It should be noted that even with nonflammable electrolyte, the cell still trigger TR and combustion, but the intrinsic safety can be effectively improved. Compared with the reference electrolyte, pouch‐type cell with nonflammable LHCE can effectively increase self‐generated heat *T*_1_ by 4.4 °C, increase TR trigger *T*_2_ by 47.3 °C, decrease the TR highest *T*_3_ by 71.8 °C, and extend Δ*t*_TR_ time by ≈8 h. Surprisingly, the pouch cell with LHCE will present a weak self‐discharge around 140 °C, which can effectively reduce the energy of cell before TR. Attributed to this unique property, the intrinsic safety parameters represented by *T*_2_, *T*_3_, and Δ*t*_TR_ can be greatly improved. Moreover, the lateral heating experiment presents that the nonflammable LHCE could increase the trigger temperature by 115.7 °C and decrease the maximum temperature of TR by 108.8 °C. In addition, the cell with nonflammable LHCE presents superior high voltage cycle stability. So far, this is the first report on the safety features of LHCE for practical pouch‐type cell. The strategy represents a pivotal step forward for practical high energy and high safety batteries.

## 2 Results and Discussion

**Figure 1:**
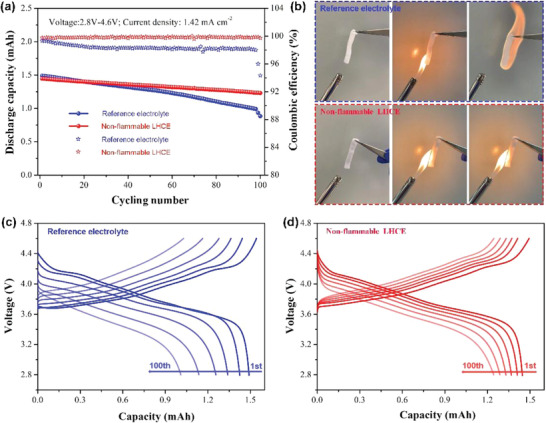
a) Cycling stability of NMC811 cells using reference electrolyte (1.0 m LiPF6/EC‐EMC) and nonflammable LHCE (1.0 m LiFSI/FEC‐TEP‐BTFE) cycled at 4.6 V. b) Flammability tests for the reference electrolyte, and the nonflammable LHCE. c,d) Charge–discharge curves of NMC811 cells using 1.0 m LiPF6/EC‐EMC and 1.0 m LiFSI/FEC‐TEP‐BTFE cycled at 4.6 V.

The harsh high‐voltage cycling (≈4.6 V) of NMC811 cells using different electrolytes is shown in **Figure** **1**. In this work, a commercial carbonate electrolyte (1 m LiPF6/ EC‐EMC, 3:7 by weight) was used as the reference electrolyte (blue curves in Figure 1a, Supporting Information), which shows obvious capacity fading (59.1% after 100 cycles) and low Coulombic efficiency (CE) of 94.34% at the end of the cycling. In sharply contrast, nonflammable LHCE presents superior capacity retention of 85.1% and a stable average CE of >99.83% (red curves in Figure 1a). Moreover, nonflammable LHCE shows more‐stable voltage profiles compared to the reference electrolyte (Figure 1), resulted from the suppressed electrolyte/electrode parasitic reactions during high‐voltage cycling. In addition, the flammability of these electrolytes was measured with ignition tests (Figure 1b): the separator soaked in reference electrolyte burns immediately. In contrast, the separator with TEP‐based LHCE infiltration cannot be ignited, attributed to the P‐based free radicals derived from TEP pyrolysis, which can effectively neutralize the H‐ and O‐based radicals required by combustion. These results prove that the LHCE exhibits significantly nonflammability than the reference electrolyte.

The safety feature of practical pouch‐type NMC811|Gr‐SiO cells was evaluated by using accelerating rate calorimetry (ARC). In order to reveal the TR characteristic of the batteries, key temperatures {*T*_1_, *T*_2_, *T*_3_} and Δ*t*_TR_ are defined.^[^ ^12^, ^13^ ^]^ Among these comparable parameters, *T*_1_ is the self‐generated heat temperature of the cell, and *T*_2_ (d*T*/d*t* = 1 °C s^−1^) is considered to be the TR trigger temperature. The time of the cell from the self‐generated heat to triggering TR is defined as Δ*t*_TR_ (Δ*t*_TR_ = *t*|_T = T2_ − *t*|_T = T1_). After this, the cell soon reaches the maximum TR temperature *T*_3_.^[^ ^14^, ^15^ ^]^ Extending the Δ*t*_TR_, increasing *T*_1_, maximizing *T*_2_, and minimizing *T*_3_ are important for practical high‐safety LIBs.

**Figure 2:**
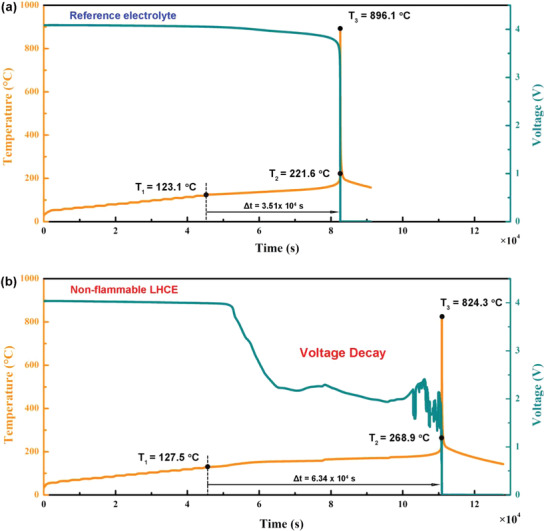
a) The intrinsic safety features of charged practical Ah‐level pouch‐type NMC811|Gr‐SiO cells using 1.0 m LiPF6/EC‐EMC electrolyte under ARC test. b) The intrinsic safety features of charged practical Ah‐level pouch‐type NMC811|Gr‐SiO cells using nonflammable LHCE (1.0 m LiFSI/FEC‐TEP‐BTFE) under ARC test.

As shown in **Figure** **2**, the practical pouch‐type NMC811|Gr‐SiO cell with reference electrolyte presents the self‐generated heat *T*_1_ temperature of 123.1 °C, and then triggers TR at 221.6 °C (*T*_2_) with Δ*t*_TR_ time of only 3.51 × 10^4^ s (Figure 2a). In sharp contrast, the *T*_1_ and *T*_2_ of the pouch cell with nonflammable LHCE increase to as high as 127.5 and 268.9 °C, respectively (Figure 2b), which reveals that the electrode–electrolyte interphase decomposition of nonflammable LHCE cell generates less heat than that of reference cell. Furthermore, the Δ*t*_TR_ time can be effectively extended to 6.34 × 10^4^ s, and the maximum TR temperature *T*_3_ can be lowered to 824.3 °C. As a result, compared with the reference electrolyte, practical pouch‐type cell with nonflammable LHCE can effectively increase self‐generated heat *T*_1_ by 4.4 °C, increase TR triggering *T*_2_ by 47.3 °C, decrease the TR highest *T*_3_ by 71.8 °C, and extend Δ*t*_TR_ time by ≈8 h. To the best of our knowledge, this is the most comprehensive safety improvement reported so far.^[^ ^10^, ^11^, ^15^ ^]^ More surprisingly, the pouch cell with LHCE presents an extremely slow open‐circuit voltage (OCV) decay from ≈140 °C, producing a self‐discharge far too weak to trigger TR, which can continuously and effectively reduce the energy of cell before TR. Attributed to this unique property, the intrinsic safety of practical pouch‐type NMC811|Gr‐SiO cell can be greatly improved.

**Figure 3:**
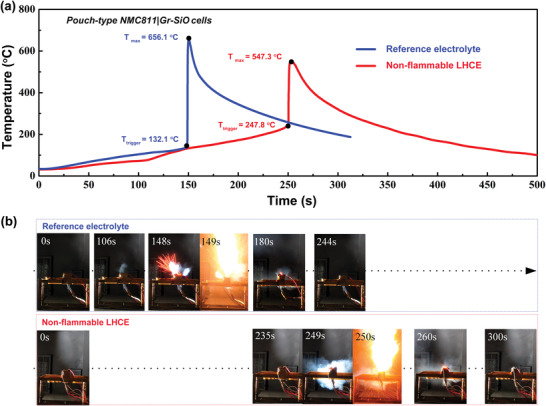
a) The external temperature of charged practical Ah‐level pouch‐type NMC811|Gr‐SiO cells using different electrolytes under lateral heating test. b) Characteristic stage of charged practical Ah‐level pouch‐type NMC811|Gr‐SiO cells under lateral heating test.

ARC can precisely measure the heat of the cell in an adiabatic environment, leading to quantifiable effective analysis of intrinsic safety. Simultaneously, the ignition characteristics of the cell can be further investigated by conducting lateral heating test in operational environment. The practical pouch‐type NMC811|Gr‐SiO cell with reference electrolyte exhibits the TR trigger (*T*_trigger_) and maximum (*T*_max_) temperature of 132.1 and 656.1 °C, respectively (blue curves in **Figure** **3**a). In sharp contrast, the pouch cell with nonflammable LHCE could achieve higher *T*_trigger_ of 247.8 °C and lower *T*_max_ 547.3 °C, respectively (red curves in Figure 3a). As shown in Figure 3b, the TR triggering time (249 s) of the cell with nonflammable LHCE is 68.2% longer than that of reference cell (148 s). In addition, the reference cell shows that continuous combustion lasted ≈32 s. In spite of the adoption of nonflammable LHCE, explosive combustion still occurred in the lateral heating test. Fortunately, the continuous combustion lasted just 10 s, effectively reduced by 68.6%. It is clear that practical pouch‐type NMC811|Gr‐SiO cell with nonflammable LHCE is considerably safer than the cell with reference electrolyte.

**Figure 4:**
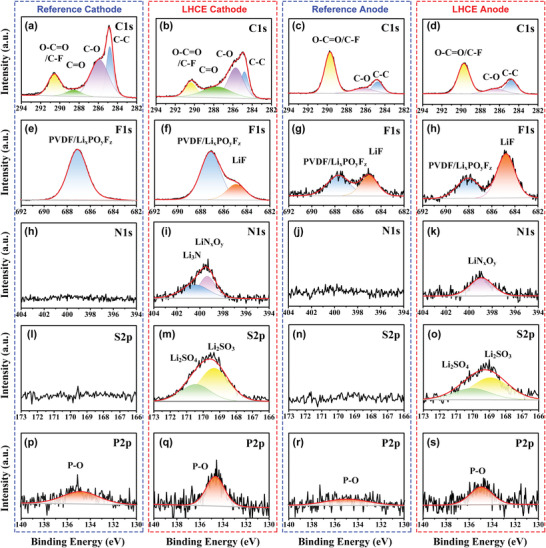
XPS spectra of C1s, F 1s, N 1s, S 2p, and P 2p for the interphase of NMC811 cycled in a,e,h,l,p) reference electrolyte, b,f,i,m,q) NMC811 cycled in nonflammable LHCE, c,g,j,n,r) Gr@SiO cycled in reference electrolyte, and d,h,k,o,s) Gr@SiO cycled in nonflammable LHCE, respectively.

The EEI (including anode–electrolyte interphase [AEI or SEI] and cathode–electrolyte interphase [CEI]) is a markedly crucial factor for electrochemical and safe properties. In order to reveal the EEI formation of NMC811 and Gr‐SiO in different electrolytes, the chemical compositions were characterized in‐depth by using XPS (**Figure** **4**). Overall, cathode and anode cycled in the same electrolyte present consistent characteristics. In detail, these surfaces of NMC811 cathode and Gr‐SiO anode cycled in the nonflammable LHCE contain less organic components, which is confirmed by the lower intensity of C 1s spectra (Figure 4a–d). Compared with the F1s of the electrode cycled in reference electrolyte, more stronger inorganic LiF signal (685 eV) could be observed in the NMC811 cathode (Figure 4e,f) and Gr‐SiO anode (Figure 4g,h) cycled in LHCE, which originates from the participation of LiFSI salt and fluorinated solvent upon cycling. In addition, the N 1s, S 2p, and P 2p spectra are used to further reveal the inorganic species. The N (Figure 4h–k) and S (Figure 4l–o) signals derived from the salt anions FSI^−^ are only found in EEI for the LHCE, which can effectively inhibit further parasitic reactions. The peak intensity of P 2p spectra of electrodes cycled in LHCE (Figure 4q,s) is higher than those of cycled in reference electrolyte (Figure 4p,r), indicating that TEP decomposition is involved in interphase formation. These results indicate that manipulating the salt/solvent chemistry of the electrolytes enables a more robust inorganic EEI on the NMC811 cathode and Gr‐SiO anode, so further helps inhibit cathode phase transformation and electrode/electrolyte parasitic reactions.

**Figure 5:**
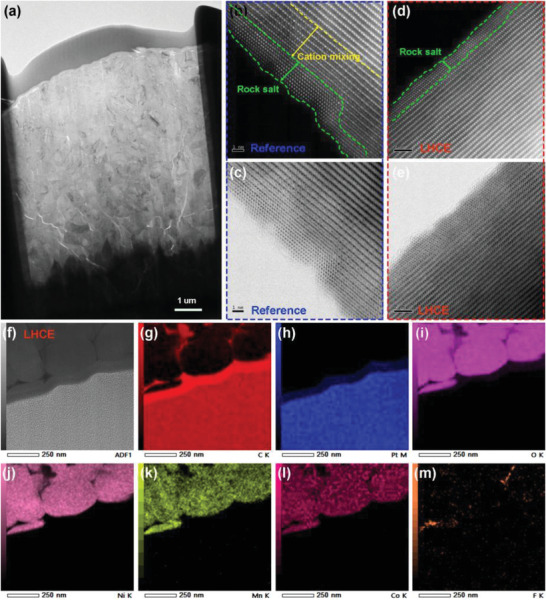
a) Image of cycled NMC811 cathode samples thinned by FIB technology for electron microscope characterization. STEM‐HAADF and STEM‐ABF images of NMC811 using b,c) reference electrolytes and d,e) nonflammable LHCE. f–m) Elemental distribution image of NMC811 cycled in nonflammable LHCE.

Deeper insight into the effective cathode protection in LHCE was obtained from STEM‐HAADF and STEM‐ABF characterization. **Figure** **5**a shows the image of cycled NMC811 cathode samples thinned by FIB technology. A thick and uneven rock salt phase layer (≈2.6 nm) is observed on the surface of aggressive cathode from reference cell, followed by a thicker (≈3.2 nm) of cation mixing layer (Figure 5b,c). These results indicate the poor surface structural stability. As a comparison, significantly enhanced structural stability of aggressive cathode can be obtained by using nonflammable LHCE, which exhibits a very thin rock salt phase (≈1.5 nm). The robust inorganic CEI generated on aggressive cathode can effectively suppress corrosion by the electrolyte and alleviate the formation of the disordered rock salt phase (Figure 5d,e). The elemental mapping shows the distribution of F elements on the surface of aggressive cathode employing the LHCE (Figure 5f–m), which is consistent with the result of XPS. The above results further confirm the significance of the stable inorganic‐rich EEI layer generated from nonflammable LHCE on efficient protection of electrodes.

## 3 Conclusion

In summary, we investigate the safety features of practical pouch‐type high energy NMC811|Gr‐SiO cells with nonflammable LHCE. It should be noted that even with nonflammable LHCE, the cell still trigger TR and combustion, but the intrinsic safety can be significantly improved. Compared with the reference electrolyte, pouch cell with nonflammable LHCE can increase self‐generated heat *T*_1_ by 4.4 °C, increase TR trigger *T*_2_ by 47.3 °C, decrease the TR highest *T*_3_ by 71.8 °C, and extend Δ*t*_TR_ time by ≈8 h. Surprisingly, the pouch cell with LHCE presents a weak self‐discharge around 140 °C, which can release the energy of cell before TR, thus effectively reducing risks of TR. In addition, the cell with nonflammable LHCE presents superior high voltage cycle stability, attributed to the formation of robust inorganic EEI. Until now, this is the first report on the safety features of LHCE for practical high energy pouch‐type cell. The electrolyte strategy represents a pivotal step forward for practical high energy and high safety batteries.

## Conflict of Interest

The authors declare no conflict of interest.

## Supporting information

Supporting Information

## Data Availability

The data that support the findings of this study are available from the corresponding author upon reasonable request.
