# Supplementary material for: Thermal Runaway of Nonflammable Localized High‐Concentration Electrolytes for Practical LiNi0.8Mn0.1Co0.1O2|Graphite‐SiO Pouch Cells
Source: Adv Sci (Weinh). 2022 Sep 8;9(32):2204059. doi: 10.1002/advs.202204059 (PMC9661853; doi:10.1002/advs.202204059)
Supplement: Supplementary file 1 — Supporting Information [file ADVS-9-2204059-s001.pdf]

ADVANCED SCIENCE
Open Access

Supporting Information

for *Adv. Sci.*, DOI 10.1002/advs.202204059

Thermal Runaway of Nonflammable Localized High-Concentration Electrolytes for Practical $LiNi_{0.8}Mn_{0.1}Co_{0.1}O_2$|Graphite-SiO Pouch Cells

Yu Wu, Xuning Feng, Min Yang, Chen-Zi Zhao, Xiang Liu, Dongsheng Ren, Zhuang Ma*, Languang Lu, Li Wang, Gui-Liang Xu, Xiangming He, Khalil Amine and Minggao Ouyang**

Thermal Runaway of Nonflammable Localized High-Concentration Electrolytes for Practical $LiNi_{0.8}Mn_{0.1}Co_{0.1}O_2$|Graphite-SiO Pouch Cells

Yu Wu,[1] Xuning Feng,[2,*] Min Yang,[2] Chen-Zi Zhao,[2] Xiang Liu,[2] Dongsheng Ren,[2] Zhuang Ma,[1,*] Languang Lu,[2] Li Wang,[3] Gui-Liang Xu,[4] Xiangming He,[3] Khalil Amine[4], and Minggao Ouyang[2,*]

[1] School of Materials Science and Engineering, Beijing Institute of Technology, Beijing 100081, China.
[2] State Key Laboratory of Automotive Safety and Energy, Tsinghua University, Beijing 100084, China.
[3] Institute of Nuclear and New Energy Technology, Tsinghua University, Beijing 100084, China.
[4] Chemical Sciences and Engineering Division, Argonne National Laboratory, Lemont, IL 60439, United States.
* Corresponding authors: fxn17@mail.tsinghua.edu.cn; hstrong929@bit.edu.cn; ouymg@mail.tsinghua.edu.cn

EXPERIMENTAL PROCEDURES

Materials. Machine-made Ah-level NMC811|Gr-SiO pouch-type cells were obtained dry (no electrolyte) from LiFun Technology (Xinma Industry Zone). The cells were transferred to an argon-filled glove box without exposure to ambient air, where they were filled with lean electrolyte (typical industry level: 2 mL electrolyte was injected into the 1 Ah pouch-type cells), and the formation of the cells was performed at C/10 under 40 °C. The control electrolyte was 1M $LiPF_6$ in EC:EMC (3:7 by volume) were purchased from Dodochem Ltd. LiFSI, BTFE, and TEP were obtained from aladdin and dried over activated molecular sieves before use.

Safety tests. An EV-ARC manufactured by Thermal Hazard Technology was utilized for thermal runaway (TR) tests. A K type thermocouple was inserted into the center of cell to measure the internal temperature, which is used to evaluate the cell TR performance. During the TR tests, the EV-ARC was operated under the heat-wait-seek mode. A heating step of 5 °C with a wait time of 15 min was performed on the ARC starting from 40 °C. The EV-ARC system would go into the exotherm mode to track the temperature rise of tested batteries and maintain the adiabatic condition if the measured temperature rate exceeded 0.01 °C·min-1.

Characterizations. FIB technology were performed to thin the NMC811 cathode (ThermoFisher Scios 2 HiVac, America). The STEM imaging of the delithiated cathodes were characterized using a Double Cs-Corrected STEM (JEM-ARM300F, Japan). X-ray photoelectron spectroscopy (XPS) (PHI Quanteral II, Japan) was conducted to evaluate the chemical composition in the prepared cathodes.
